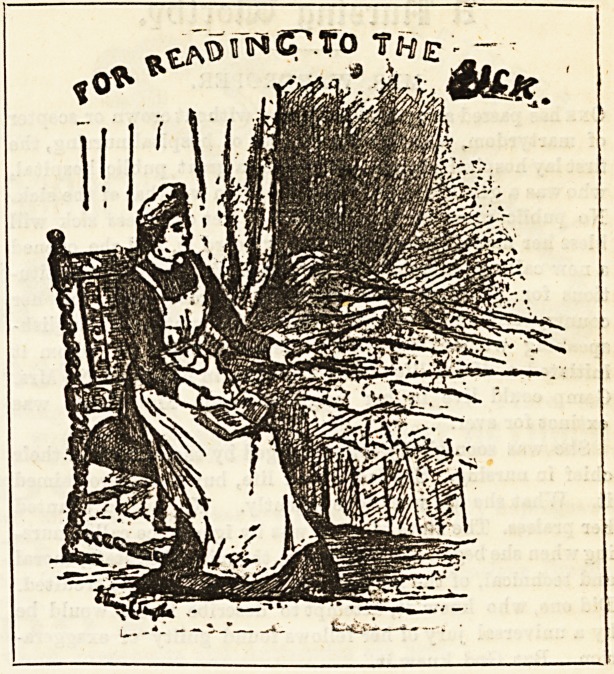# The Hospital Nursing Supplement

**Published:** 1892-12-31

**Authors:** 


### "The Hospital" Nursing Mirror.

Being the Extra Nursing Supplement of "The Hospital" Newspaper.

[Contributions for this Supplement should be addressed to the Editor, The Hospital, 140, Strand, London, W.C., and should have the word "Nursing" plainly written in left-hand top corner of the envelope.]

### En Passant.

ARBROATH DISTRICT NURSING ASSOCIATION.—A report of the first nine months' work shows the amount of work which one woman can achieve. Miss Forsyth, Queen's Nurse, has undertaken one hundred and fourteen cases and paid 3,252 visits. Macintoshes, water-pillows, and every needful appliance have been given by those interested in the work, and every society in the neighbourhood seems to have found some funds for it. Scotland certainly appreciates the development of district nursing.

COTSWOLD BENEFIT NURSING ASSOCIATION.—The first report of this society shows five cottage nurses at work, two of whom have served the Association for one year, two for nine months, and one for six months. During that time they have nursed 37 cases in addition to casual work. Five cases only have resulted in death. The Association is to be largely increased by the inclusion of three more parishes, and a new district nurse for it is now training at St. Mary's, Plaistow. There is a deficit on the year's accounts of seventeen pounds.

SHORT ITEMS.—Miss Rutherford, from the Scottish Branch of the Q.V.J.I.N. has been appointed District nurse for Blairgowrie.—The miners of Seaham Colliery have decided to have a nurse to themselves, so there will be one nurse at work at Seaham Harbour and one at New Seaham, to both of which undertakings Lady Londonderry has promised assistance.—A district nurse is to be provided for Anstruther and the neighbourhood.—Kingston District Nursing Association has received the sum of thirty pounds, the result of a sale of work held by the Honorary Secretary, Mrs. Hopkins.—Lewes is to have a district nurse.—The *Young Woman* for December contains an article on nursing as a profession for women.

EDINBURGH ROYAL INFIRMARY.—The new Nurses' Home opened on the 19th instant by the new managers of the institution is situated on the west side of the main corridor connecting the surgical and medical wings. It is two storeys high, and contains separate rooms for 101 nurses, and three sick rooms. There are sixteen baths in the building, and the large recreation room is beautifully decorated and furnished. The suggestion of the new Nurses' Home originated from the idea of the late Superintendent, Surgeon-General Fasson. The nurses and their Matron, Miss Spencer, are delighted with their new home, and after the opening ceremony, when the keys were handed over, they thanked the managers for their kindness in providing such excellent accommodation for their comfort and welfare.

ST. LUKE'S HOME, VANCOUVER.—The report of the year's work in this Home has just reached us. Sister Frances and her two nurses have been kept busily engaged, and besides doing the work of the Home they have nursed one hundred and fifteen cases, eighty-three of these being patients in the Home, while the remaining thirty-two were nursed at their own homes. This record includes eleven cases of small-pox nursed at Howe Sound. There was nobody on the spot to help when this outbreak occurred, so St. Luke's Home came to the rescue. We are glad to hear that the Convalescent Home at Chilliwack is slowly commencing to prosper. The funds of this Home are quite distinct from those of St. Luke's and come from other sources.

LONDON DIOCESAN COUNCIL FOR PREVENTIVE WORK.—We should like to draw attention to the objects of this Council, which are, perhaps, not as well known as might be, and seem worthy of support, which, however, does not appear to be adequate. The Council, which is under the immediate direction of the Bishops of London and Marlborough, endeavours to bring into union existing agencies in the diocese, and to aid them in developing their resources. It also makes grants of money to institutions and towards the support of mission women. Those who wish to aid it can do so by sending donations to the Secretary, the Church House, Dean's Yard, S.W.

MANCHESTER AND SALFORD SICK POOR AND PRIVATE NURSING INSTITUTION.—The latest report of this institution is not quite so satisfactory as its friends could wish. There is a pressing need for a greater number of annual subscribers, for the subscription list has shown an unpleasant diminution owing to the deaths of some wealthy friends. Want of funds has prevented the committee from extending the operations of the institution in a direction pointed out as desirable many months ago. Almost the only satisfactory note in the report is the statement that an advance has been made by increasing the staff of nurses, and introducing district nurses to quarters of the town which have hitherto been without their services. Beyond this the committee have been unable to extend their operations, and the scheme to provide nurses for those who cannot afford to pay for a private nurse, and at the same time are ineligible for the unpaid service of a district nurse, must remain in abeyance till the public come forward and help the institution to set the scheme afloat. There appears to be a real want for such a class of nurse, and it is to be hoped the committee will yet be enabled to meet it. During the year the district nurses attended 4,470 cases, paying 105,140 visits altogether. A fourth home is much needed as a nursing centre for North East Manchester, and want of funds alone stands in the way of establishing it.

THE SCOTTISH BRANCH OF THE QUEEN VICTORIA JUBILEE INSTITUTE.—The necessity of a speedy addition to the Home, owing to the increased demand for nurses both in Edinburgh and in county districts, is pointed out in the fourth Annual Report of this Institute for Nurses. The same report states that during the year fourteen new local associations have been formed in connection with the Institute, and have engaged Queen's Nurses. Nineteen hundred and sixty-nine cases have been nursed, and 40,977 visits have been paid during the year. In order to provide for further and much-needed accommodation, two flats above the existing Home have been purchased and partly furnished. By this means twenty-three nurses can be accommodated, and room can be provided for more if necessary. Twenty-seven district probationers have entered the Home during the year, and twenty nurses have completed their training and received appointments. The nurses in the Home consisted of one superintendent, one district superintendent, one assistant, and three permanent nurses; thirteen were undergoing six months' district training, one was training in Chalmers' Hospital, and one in Leith Hospital, eight in Edinburgh Royal Infirmary, and two in Barnhill Hospital, Glasgow. A most satisfactory feature of the report is the substantial increase shown in the amount received, not only from donations, but from subscriptions. The Pension Fund for Queen's Nurses in Scotland, which was started last year with a gift of £500, now amounts to over £800.

### The Development of Children by Gymnastics.

II.

Having spoken generally of the importance of physical exercise for the young, I wish to notice more particularly the good to be derived from the various exercises, dividing the subject into three groups, viz. : (1) General exercises with apparatus adapted for the school-room. 2. Gymnastic exercises. 3. Swedish drill. The first of these headings will embrace those exercises performed with (a) the bar or wand, (b) dumb bells, (c) Indian clubs. I take these as being the ones most generally used in our school and home drill, and as being the characteristic apparatus of the many kinds to be obtained. They are quite sufficient for ordinary school drill, and should further exercise be sought, the gymnasium will furnish all else to be desired.

A great obstacle to children joining regular drill classes seems to be the dress. Many people object to providing a special costume. This is not absolutely necessary with the first group of exercises, as long as the limbs can move freely, and in the present style of children's dress this is, happily, generally the case; but, with a number of pupils, it is well to secure the appropriate dress of each by issuing an order that a jersey body at least should be worn, and this does not necessitate change of costume before and after drill. If we wish to have a "show" class, it undeniably adds to the effect of the exercises if all the members are in costumes of the same colour and pattern, and as these have to be specially made, we secure a dress which is properly adapted to free movement of all the limbs.

Of course it is desirable to derive as much good as is possible from these and every exercise, and in order to do this we must pay strict attention to—(1) the time of exercising; (2) position; and (3) the manner of execution.

The time of these classes is by no means unimportant. If possible it is well to leave from two and a-half to three hours between a meal and the exercises which follow, but if this be not convenient, at least one hour should intervene.

The position of the pupil is perhaps the most important point to be insisted upon. The child should stand perfectly upright with head erect and chin up, shoulders well back, arms hanging close to the sides, while the waist is drawn in, heels together and feet at right angles. Very careful notice should be taken throughout the class that this position is maintained, and, immediately the slightest tendency to a careless posture is observed, a sharp command to "attention" given. With children this is even more necessary than with adults, their muscles being undeveloped, and the whole body more pliant, so that exercises in a wrong position will often tend to produce deformity, such as round shoulders, contracted chests, and the injurious and unsightly "stork-habit" of standing on one leg with the hip stuck out.

A good teacher will endeavour to interest his pupils in the exercises, for it may well be understood that, to any intelligent being, a series of movements made without apparent rhyme or reason will, after the first sense of novelty has worn off, become irksome, and briskness of execution and "finish" will be impossible to obtain with such a class. It is essential to keep the mind in constant play, so that is is well to give a few clear, short commands before the pupils attempt each exercise, that they may be prepared for what is coming, and give more attention to the execution. A knowledge, also, on the part of the pupil as to the particular muscles to be developed by the exercise in progress will enable him to intelligently go through the drill.

Turning now to the exercises themselves. The pupils are standing at "attention," with the arms hanging close to the sides, and each clasping the bar firmly in both hands, and dividing it into three equal parts. In the first the rod is raised forward slowly above the head as high as possible (as shown in the figure), and then brought back to the starting position. In the second exercise the bar is raised above the head, and then placed on the back of the shoulders, raised again above the head, then brought back to the first position. In the third exercise the rod is raised as in the first, but the hands are allowed to slip along the pole towards the ends (this must be done in one movement while raising the arms), then the rod is dropped at the back till the arms are fully extended; the rod is then brought back to position in two movements. These are the primary exercises of this group, and may of course be extended and varied; their chief object is to expand the chest, exercise the arms, and work the "deltoid" or shoulder blade muscles. In addition to these arm movements we may at the same time use a profitable leg exercise. When the rod is lifted rise on the ball of the foot and sink again as it is lowered. This serves to strengthen the muscles of the calf and foot, and by combining it with the arm movement time is gained for other exercises.

The next exercises to be noticed are those which bring into play the "rotator" muscles. For the first of these the rod is raised horizontally above the head, then the body bent, from the waist only, first to the right, at the same time bringing the rod to a perpendicular position as far to that side as possible. After this, the same movement is made to the left, and the arms brought over to that side.

For the second exercise, stand at "attention," with the rod held in front (as in exercise 1), then swing the arms up above the head to the left side till the upper part of the body is facing round to the left; then, making a half-circle in front of the body with the rod, raise it to the right side, and *vice versa*.

In exercise three, starting as usual from "attention," raise the arms in front to the level of the shoulders, keeping them well extended, then, without moving the feet, turn slowly to left and then to the right till the command to stop is given. This movement is particularly good for strengthening the ankles. These three exercises are most effectual in cultivating grace and elegance of figure and movement, and this is increased when done in waltz time to a musical accompaniment.

Before leaving the bar exercises we must notice one which is capital for cultivating brisk motion of the arms and shoulders, and is very good as a correction to "round backs." The hands are slid along the bar till they are within a few inches of the ends, the bar is then raised and placed behind the head well down on the shoulders; keeping a firm grasp of the rod, the left arm is extended sharply to its full length, and the right arm brought up to the right shoulder in a similar way, the right arm is then extended and the exercise repeated. This is done to a moderately quick march time. It expands the chest, besides working the muscles of the arms; it makes the wrist strong and flexible. When each set of exercises is finished it is well to train the children to "fall in" and march off in orderly manner, as this does much towards promoting prompt obedience to orders, and will be found a great assistance in management of the class, especially when the numbers are great.

### Nursing Homes.

### VIII.—ROYAL FREE HOSPITAL.

The Royal Free Hospital is hardly more than a name to people inhabiting the West or the East Ends of London, but in its own immediate neighbourhood it is really a very important institution. It stands exactly where it is wanted most, right in the midst of a densely-populated region, and the inhabitants of this district certainly show their appreciation of it most practically, for they keep it full! There are no closed wards and empty beds at the Royal Free—indeed, it needs good management to ensure accommodation being always available for such "urgent cases" as are admitted "at all hours of the day or night" to general hospitals.

Entering through the great doors which are promptly opened to receive all comers we find ourselves in the courtyard, which is a special characteristic of the place. The frontage in Gray's Inn Road is to be rebuilt ere long, as it is not only inconvenient, but in very bad condition structurally But we trust this will not deprive the patients of even one square yard of their popular airing-ground. It is really a pleasant spot, with trees and garden-seats for such convalescents as are "allowed out" by their doctors. There is much coming and going through the square, and that "keeps us lively" say the invalids, and the open space also allows light and air to find their way into the blocks of wards where so many sick and suffering men, women, and children lie patiently in their beds. The whole place is surprisingly cheerful and bright looking, forming a delightful contrast to the neighbouring streets which have neither of these attributes in most people's eyes.

Passing through the sunny courtyard and underneath an archway we reach the Nurses' Home, which occupies a quiet corner in the pile of buildings which represents "a poor hospital in a poor neighbourhood"—a hospital, moreover, where a large amount of excellent work is achieved in the course of each year, and which ought, therefore, to be liberally supported and freed completely from that "urgent need of funds" from which it chronically suffers.

The cubicles where the staff nurses sleep line each side of the long, bare dormitory, and seem of a very plain and homely character when considered as the accommodation provided for "charge nurses," who hold much the same position as the "ward sisters" in other hospitals, and are women of ability and experience. The probationers' cubicles are similar, and are situated below the nurses', and the latter, as well as the former, are provided with a small sitting-room, but the bath-rooms are insufficient and inconvenient. Also there is such very limited accommodation that if an emergency necessitated two or three additional nurses, it would be impossible to engage them, however urgently the work might demand their services, on account of the lack of room. All people conversant with the vicissitudes of systematic nursing will realise the inconvenience of such straightened quarters. The meals for the nurses are served in a large downstairs apartment, where one or two pretty plants give a somewhat festive look to the long tables.

We should be glad to think that the day is not far distant when a handsome gift from some philanthropist, who has nurses' welfare at heart, will render it possible for this useful little hospital to be supplied with a convenient and fitting "home," in which shall be plenty of space and suitable furniture, and other comfortable surroundings liberally provided for all. In the meantime it is pleasant to find a cheerful spirit animating the workers and inspiring them to make the best they can of their present inadequate quarters, to which, possibly, use has to a great extent reconciled them, although we cannot doubt the willingness with which the ugly dormitory would be resigned and the noisy cubicle exchanged for a cosy little separate bed-room which would assume some of the attributes of home.

In designing a fine new frontage and in planning for convenient offices, and for the sorely-needed casualty department, let the liberal public remember what it owes to the nursing staff, and cheerfully provide the funds so pressingly demanded.

The Nursing Home should not be the last item on the list of necessaries, and good workers certainly merit proper housing. In consideration for the comforts of the patients, we think the Royal Free Hospital can justly congratulate itself on its achievements, and we do not think it will long be content to let the convenience of those who minister to the sick be passed over and disregarded.

### Novelties for Nurses.

To many other useful personal equipments Messrs. Allen and Hanbury have lately added a nurse's testing case. It is neat, small, and convenient in dimensions, and contains all the necessaries for the ready examination of urine. Messrs. Allen and Hanbury, Plough Court, Threadneedle Street, and Vere Street, Cavendish Square.

---

An excellent bandage roller has been brought out by Bellaers, of Leicester, which will soon become a great avourite with nurses. It is easy to work, light to carry, and yet perfectly steady when screwed on to any ordinarily firm table. In addition to its other virtues, it possesses that of cheapness, and can be bought for 6s. 6d. without a case, and for 9s. in a neat japanned box, very convenient for carrying. Address of maker: James Bellaers, Regent Street, Leicester.

### Having or Giving?

Christmas and gifts are two words which certainly pair together very pleasantly and naturally. Whilst some people have it in their power to give, others are glad to receive, and both processes are decidedly agreeable. Perhaps of all places in the world hospitals are those where Christmas gifts are most appropriate. Few people who have never lived within the walls are able to realise the actual extent of the joy which can be thus secured to the sick and suffering. A great many poor souls, spending the festival in a ward for the first time, experience an amount of pleasure difficult to estimate merely from being the recipient of a personal gift, something both useful and nice to look at, and to feel. By the way, what observant person can fail to note the appreciative touch with which the hand, unaccustomed to such things, lingers about the soft texture and warm materials? The warded patient has out the "Christmas box" many times during the day, and looks and fingers admiringly the welcome and novel possession. We like to think of the presents recently provided by readers of The Hospital being thus treated.

Gifts to the wealthy are hard to choose, because such folks have so many good things already! But for those who have very small possessions it becomes an easy matter to furnish suitable gifts. With regard to the grand Christmas trees just now in fashion at those institutions where the pleasures of patients are fittingly considered, there is much which might be said with advantage. When special donations allow (for, of course, no one would wish the maintenance funds thus expended), it is good to hear of seasonable gifts for nurses and servants being displayed alongside of those intended for the patients, but, of course, this cannot always be done, however much the officials might wish it. Yet, when possible, it is a kindly practice which gives pleasure to all.

However, there is another very different custom, we regretfully observe, at some places where, instead of the staff of earnest workers, outside visitors are the recipients of the gifts from the ward trees. A curious impression seems to prevail that because toys are provided for sick and poor children, those little people who form always a proportion of the guests, should likewise receive presentations. Surely the lesson thus taught is a bad one? If the well fed and healthy, smartly-dressed little ladies and gentlemen knew it as an invariable rule that the public trees were only for their poor little brethren, they would learn to see without coveting the gay-coloured gifts.

Children are easily led, and they might quickly be taught that "having" is not the chief thing to be aimed at. A group of handsomely clothed bairns leaving a hospital gate laden with toys makes but a sorry sight; and the same small guests would quit the doors with equally bright looks on their sweet little faces if parents and guardians would but give themselves the trouble to teach the easily-mastered lesson, that there is a good time for "giving" as well as for "having" even in the daily life of a little child.

### Wants and Workers.

The members of an Amateur Dramatic Club would be pleased to give a gratuitous performance in aid of a hospital or charity in London or the suburbs, or for the amusement of patients. The Hon. Secretary will be glad to give further particulars if applied to by letter at her address, 11, Selwood Place, Onslow Gardens, S.W.

*Sister Lucie, Littleboro,* begs to acknowledge with many thanks the parcel of old linen from Bayswater, sent anonymously.

*A Home for the Cure of Hysteria.*—We have received three replies from medical men and twenty-six replies from nurses and nursing homes in answer to "St. Helena," and have forwarded them to her. From the terms quoted in many of the letters, we fear that some of our correspondents have not understood that appeals of this sort are *only* inserted in our columns in the cases of persons whose poverty or great need makes the ordinary fees charged an impossibility, and who would only be able to pay the most moderate sum for the care given them.

*Home for an Inebriate.*—We have received four replies, in answer to "Nurse," which we have forwarded.

*Home for an Epileptic.*—One reply has been received to this appeal, and we have forwarded it.

### Everybody's Opinion.

[*Correspondence on all subjects is invited, but we cannot in any way be responsible for the opinions expressed by our correspondents. No communications can be entertained if the name and address of the correspondent is not given, or unless one side of the paper only be written on.*]

### NURSES' FEES.

J. E. writes: I should like to say a few words on the subject of reduced fees for those who cannot afford to pay the very highest class of nurses. There are many women who undoubtedly served the sick and afflicted well, long before this craze of recently trained nurses took such entire possession of the public mind. The "trained nurse" has become a trite subject; why not take up the case of their predecessors, if only by way of a change? There is a large class of nurses, of from forty to fifty years of age, highly respectable women, who, because they are getting on in years, and because, though capable and experienced, they have not had what constitutes the modern idea of "training," cannot get taken on at a good institution, or should they have been taken on at some time or other, and have left through illness, find getting on again very much of a difficulty. Such nurses, and there are many of them, would gladly go out at reduced fees, or even half the fees usually charged at an institution. These institutions are gradually disappearing, but meanwhile it is hard times for those who only have their old profession to look to for a livelihood. If in your good Hospital you would suggest some scheme for the assistance of this class of nurse, it would be a good thing done for both sides; if some place could be found where nurses, duly proved capable, could register their names, and where patients who can only afford to pay the most moderate wage to a sick nurse, could find themselves brought into relation with those willing and able to serve them, it would be the greatest boon. I know there will be many differences of opinion on such an idea, but I venture to think some of the old hands are as able to take a case as some of the new ones.

### THE LATEST HOSPITAL AUTHORITY.

Mr. H. S. Alexander writes from Darmstadt under date 20th inst.: We wished to be quite courteous, but why does not our correspondent either deny or admit the correctness of the answer given on page xci. of the Mirror for 17th inst.? He seems to be unaware that the charges in question were published over his name so recently as the 8th inst. The letter is as follows:—

Sir—As your answer to a query *re* myself in your issue of the 17th inst. borders on, apparently, intentional discourtesy, perhaps you will insert these few lines. If the person who hides him or herself under the pseudonym "Curious" will communicate with me, I shall be happy to answer the query put more correctly than you seem to be in a position to do. I suppose the query comes from some distant country, for what are termed my "charges" appeared such an age ago that if they were "stale" they must have been by now long forgotten by those who may have taken the trouble to read them. Being quite unknown in London, my age and abilities having so far prevented my pushing myself into notoriety, as other people have done, by taking up the criticising of hospitals as, one may almost say, a profession, I cannot understand how, except by a process of fabrication, you arrived at the information contained in your ridiculous answer to the query put by "Curious." Perhaps "Curious" will also kindly explain to me how charges can "have so familiar a ring as to appear stale from repetition"? If whatever this elaborate statement is intended to convey is correct, why did "Curious" take the trouble to send you his query?

### Presentations.

The Slaithwaite Women's Co-operative Guild has presented the Matron of the Huddersfield Nurses' Home with a very handsome silver fruit basket, in return for seven lectures on Nursing.

Nurse Constance Thomas, on the completion of her course of lectures for the Middlesex County Council at Teddington, was presented with a handsome travelling bag, an ivory-backed brush and comb, a bouquet of flowers, and a letter of thanks.

### Appointments.

[It is requested that successful candidates will send a copy of their applications and testimonials, with date of election, to The Editor, The Lodge, Porchester Square, W.]

Derby Royal Infirmary.—Miss S. Carvasso has been appointed Matron to this infirmary. Miss Carvasso trained at St. George's Hospital, London, where, after her training, she held the appointment of head nurse, and temporarily that of matron and night superintendent. Miss Carvasso is at present the Matron of Salisbury Infirmary, where she has done most excellent service, and will be greatly missed. We congratulate the Committee at Derby on this appointment.

Old Machar Poorhouse.—Nurse Marion Hay, who, six months ago, completed her three years' training at the Aberdeen Royal Infirmary, has just entered upon her duties as head nurse to this institution. Nurse Hay leaves her "Alma Mater" with the cordial good wishes of all her fellow-workers.

### Minor Appointments.

[We propose to insert from time to time under this heading the appointments of Assistant Matrons, Charge and Staff Nurses, if our readers will kindly keep us informed of their promotion.]

Bath Royal United Hospital.—Nurse Hamilton, who trained at this hospital and has since been charge nurse of the Helena, Katherine, and Maud Wards for three years, has been made charge nurse of the Albert and Leopold Wards (men's surgical); Nurse Ada Boron, who trained at the Royal United Hospital, has been appointed charge nurse of the Duncan and Edinburgh Men's Medical Wards; and Nurse Rose Fenemore, who was also trained at this hospital, has been appointed charge nurse of the Helena, Katherine, and Maud Women's Surgical Wards.

### Notes and Queries.

### Queries.

(37) *Book Wanted.*—Where can I get a copy of the "Human Body," by Owen Lankester?—*A. W.*

(38) *Psoriasis.*—Please give me some hints as to how to treat "psoriasis?"—*Sister Dora.*

(39) *Bedsores.*—Can anyone tell me of anything I can use for an old and partially-paralysed person, who, from constant sitting, has her skin broken and rubbed?—*K. M.*

(40) *Nursing in Australia.*—Kindly inform me to whom I should apply for an appointment, either in hospital or private institution, in Melbourne or Sydney?—*Carlisle.*

(41) *Throat Deafness.*—I have been advised to obtain an audiphone for a case of throat deafness. I am anxious to know the maker's name? —*Brisbane.*

(42) *Where to Lodge.*—Will you kindly tell me where a nurse can lodge while staying in London for a fortnight?—*Country Bumpkin.*

(43) *R. N. P. F.*—Where can I obtain a prospectus of the Nurses' Pension Fund? Also tell me a good nurses' manual and dictionary?—*Eric.*

(44) *How to Pass the L.O.S.*—Where can a trained nurse, going out as a missionary, train in midwifery, dispensing, and district work? *Denbigh.*

(45) *Where to Lodge.*—Where can I put up for a short time?—*H. M., Dorset.*

### Answers.

(34) *Midwifery at Birmingham* (*Nurse Charlotte*).—Write to A. F. N., 69, Friar Street, Warwick, and see if she can help you.

(37) *Book Wanted* (*A. W.*).—Correspondents must really look in the paper before asking needless questions. The "Human Body" was reviewed in The Hospital for December 17th, page 188; and it was there stated that Allman and Sons were the publishers.

(38) *Psoriasis* (*Sister Dora*).—Please consult a medical man and not a newspaper on such a point as this.

(39) *Bedsores* (*K. M.*).—If your patient has not already a bedsore she very soon will have. Consult a medical man at once.

(40) *Nursing in Australia* (*Carlisle*).—You must apply direct to the matrons at the hospitals. Do not go out on chance, as the Colonies now train so many of their own nurses. You will see all particulars of the best hospitals and institutions in Australia in the book, "How to Become a Nurse," published 1d. at this office.

(41) *Throat Deafness* (*Brisbane*).—There are many kinds of audiphone, any of which can be obtained from a surgical instrument maker, but we advise you before obtaining one to consult an aurist.

(42) *Where to Lodge* (*Country Bumpkin*).—We should advise you to go either to Miss Culverhouse, 18, Royal Avenue, Chelsea, or to the Nurses' Residential Club, 92, Charlotte Street, Fitzroy Square.

(43) *R. N. P. F.* (*Eric*).—Write to the Manager, 8, King Street, Cheapside, for particulars of Pension Fund. Get Lewis's "Theory and Practice of Nursing" and "The Nurses' Dictionary" from this office.

(44) *How to Pass the L.O.S.* (*Denbigh*).—You cannot do better than write to Sister Katherine, St. Mary's Nurses' Home, Plaistow, East.

(45) *Where to Lodge* (*H. M., Dorset*).—Your letter received too late to reply by post. See answes to "Country Bumpkin."

### THE NEW YEAR.

The rejoicings of Christmas are nearly over, and what the New Year, which draws so close, may bring us is full in our thoughts. Let us stop a moment, however, and recall the past one. Did 1892 bring us health and happiness, or sickness and disaster? A mingling of each most likely, for misfortunes only come now and then, a bad accident one year, a fit of sickness another, or in a third the loss of one most dear, which robs life of its brightness for many a day. These are the real trials of life, for when people complain that everything goes against them, we suspect that they are themselves perverse and go against everybody, while their carelessness or inattention will prevent success in life. Happiness, on the other hand, is so continuous, made up of so many little things, that we are frequently unable to recognise it is happiness. There is, for instance, the comfort of a home over our heads, with sufficient food and clothing, even if they are not of the choicest; but who counts these up as mercies; are they not our rights? I am afraid many of us think so. Then there are our kind relations and friends, our innocent amusements, unexpected presents, difficulties smoothed in our paths; all these things make happiness if we will own it, while a word of praise bestowed on our work by one who seemed hard and austere sends a thrill of joy through us and comforts our hearts. Our own kind actions and the putting ourselves out of the way to oblige others are not among the least of our causes of happiness, but if we have what the old folks called "the root of the matter" in us, *i.e.*, that our actions are guided by love and duty towards God, all ordinary rubs and troubles pass over us like water from a duck's back. Some call troubles the "visitation of God." Oh! would that we could fully realise that He visits us at all times, that He is about our bed, and about our paths and spieth out all our ways, that He gives His angels charge over us to keep us. Shall we not begin the New Year with a cheerful spirit, determined to try and make the best of whatever befalls us. We will be thankful for our good things, taking them and our trials as coming alike from Him who knoweth what is best for us. Sickness and troubles become unbearable when we are always thinking of them and bemoaning our fate, while a merry, cheerful heart doeth good like a medicine. It is well if we know this, but the old psalm quaintly says—

"And know what's right, nor only so,
But also practice what you know."

In following this rule we shall go a long way towards making 1893 a happy year.

### A Nursing Worthy.

MRS. WARDROPER.

One has passed away without noise, without crown or scepter of martyrdom, who was the pioneer of hospital nursing, the first lay hospital matron, at least of a great public hospital, who was a gentlewoman. Her kingdom was that of the sick. No public press heroine was she. Yet countless sick will bless her name, though they never heard it, and she opened a new calling for women of all classes, the nursing in institutions for the poor. She did this, a great work, for her country and her sovereign, who is the mother of English-speaking womankind—thrice blessed to those for whom it initiated a divine life of common sense in nursing. No Mrs. Gamp could live in her neighbourhood. Mrs. Gamp was extinct for ever.

She was soon gladly acknowledged by the doctors as their chief in nursing. She led a hard life, but never proclaimed it. What she did was done silently. No herald chanted her praises. The state of what was by ignorance called nursing when she began hospital work, the miserable state, moral and technical, of the nurses, would scarcely now be credited. Did one, who knew it, attempt to describe it, she would be by a universal jury of her fellows found guilty of exaggeration. But God knew it.

I saw her first in October, 1854, when the expedition of nurses was sent to the Crimean War. She had been then nine months matron of the great hospital in London, of which for 33 years she remained head and reformer of the nursing. Training was then unknown. The only nurse, worthy of the name, that could be given to that expedition, though several were supplied, was a "sister" who had been pensioned some time before, and who proved invaluable. I saw her next, after the conclusion of the Crimean War. She had already made her mark. She had weeded out the inefficient, morally and technically. She had obtained better women as nurses. She had put her finger on some of the most flagrant blots, such as the night nursing. And where she laid her finger the blot was diminished, as far as was possible. But no training had yet been thought of.

All this led to her being chosen to carry out in the hospital of which she was matron the aims in the training of nurses of the Nightingale Fund, which had then been subscribed. She was named first superintendent of that school, and continued such for twenty-seven years, till her retirement in 1887. That school under her has been more or less the model of all the subsequent nurse-training schools, which now nearly every considerable hospital, and many an inconsiderable, has its own. But they chiefly train for themselves. She, as head of the Nightingale School, trained for many other hospitals and infirmaries.

The principles of this school may be shortly said to be as follows: (1) That nurses should have their technical training in hospitals specially organised for the purpose; (2) that they should live in a home fit to form their moral life and discipline. The school under this lady was opened at the old St. Thomas's Hospital, near London Bridge, in 1860. St. Thomas's and the Nightingale School were removed to the Surrey Gardens in 1862, and in 1870 to their present abode opposite the Houses of Parliament on the other side of the river.

At the time of her retirement upwards of 500 nurses had completed their course in the school, and entered into service on the staff of St. Thomas' and other hospitals, and of these over fifty educated gentlewomen were occupying important posts as matrons or superintendents of nurses in hospitals, infirmaries, and nursing institutions for the poor, and not only in the United Kingdom, but also abroad. This lady was Mrs. Wardroper, who passed away, quite peacefully, in her eightieth year, on December 15th, 1892, and was buried quietly on December 19th.

It is difficult to describe the character of such a woman, the more so as her praises were never sounded in newspaper or book. No laurel encircles her brow, no crowds assemble to do her honour. But hundreds of trained nurses and hundreds of thousands of well-nursed sick are her meet tribute. Her power of organisation and of administration, her courage and her discrimination in character were alike remarkable. She was straightforward, true, upright. She was decided. Her judgment of character came by intuition—at a flash. Not the result of much weighing or consideration. Yet she seldom made a mistake. And she would take the greatest pains with her written delineations of character required for records, writing them again and again, in order to be perfectly just, not smart or clever; but they were in excellent language.

She was free from self-consciousness. Nothing artificial about her. "She did nothing because she was being looked at, and abstained from nothing because she was looked at." Her whole heart and mind, her whole life and strength were in the work she had undertaken. She never went a pleasuring, seldom into society. Yet she was one of the wittiest people one could hear on a summer's day, and had gone a good deal into society in her unmarried life. She was left a widow at forty-two with a young family. She had never had any training in hospital life. There was none to be had.

Her force of character was extraordinary; her word was law. For her thoughts, words, and actions were all the same. She moved in one piece. She talked a great deal, but she never wasted herself in talking; she did what she said. Some people substitute words for actions, she never. She knew what she wanted, and she did it. She was a strict disciplinarian; very kind, often affectionate, rather than loving. She took such intense interest in everything, even in things matrons do not generally consider their business, that she never tired. She would be quite late in decorating the chapel at Christmas and Easter with her own hands. She had great taste, and spent her own money. She was a thorough gentlewoman—nothing mean or low about her—magnanimous and generous, rather than courteous.

And all this was done quietly. Of late years the great nursing work has been scarred by fashion on one side and by mere money-getting on the other—two catastrophes sure to happen when noise is substituted for silent work. Few remember her in these express-train days, dashing along at sixty years in a day.

> A perfect woman, nobly planned
> To warn, to comfort and command.

"Comfort," not in the present meaning of comfortable, easy-chair life, but *comfort* in the good old meaning of "Be strong with me."

And so, dear Matron, as thou wast called so many years, we bid thee farewell, and godspeed to His higher world; not as the world giveth, giveth He thee. F. N.

### The Nurses' Bookshelf.

ADVICE TO WOMEN.*

We have received from Messrs. Cassell, "Advice to Women," by Florence Stacpoole, a handbook on the care of the health before, during, and after confinement, which gives simple directions as to what to do on these occasions. There is a good chapter on the necessity of understanding the value of good drainage and antiseptic measures, and the list of requisites will also be found useful.

OUR SICK, AND HOW TO TAKE CARE OF THEM.*

From the same publishing house, and by the same author, we have received this book of "Plain Teaching on Sick Nursing at Home," which the author states is not meant for those who can afford to pay for the help of a trained nurse. There are chapters on the sick-room, and what to do in the sick-room, and many useful hints for the use of the amateur nurse, and simple recipes for sick-room cookery and the method of peptonising milk and beef tea.

* "Advice to Women," "Our Sick, and How to Take Care of Them." Two handbooks by Florence Stacpoole. (Cassell and Co.)

## Figures and Tables

**Figure f1:**
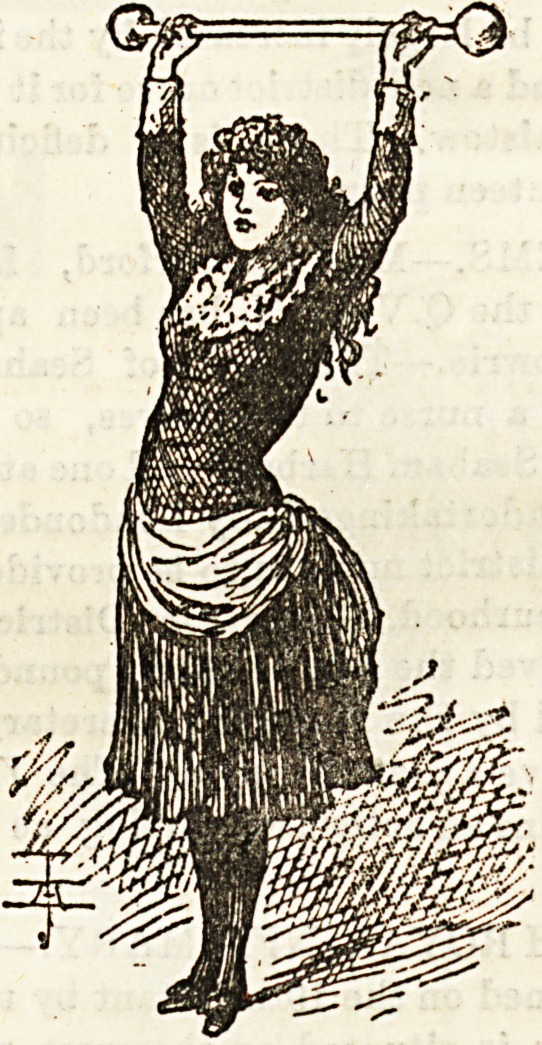


**Figure f2:**
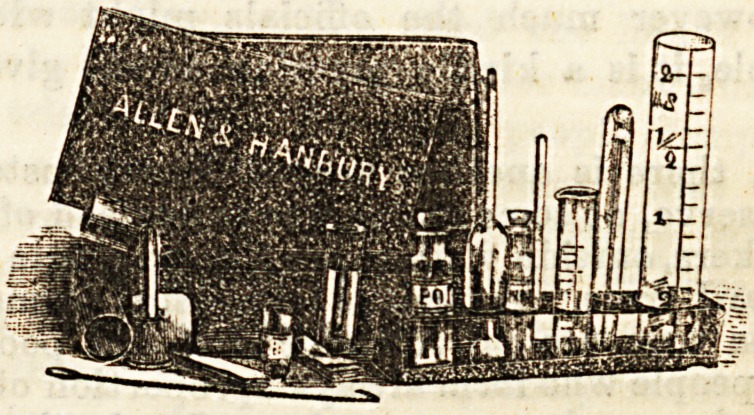


**Figure f3:**
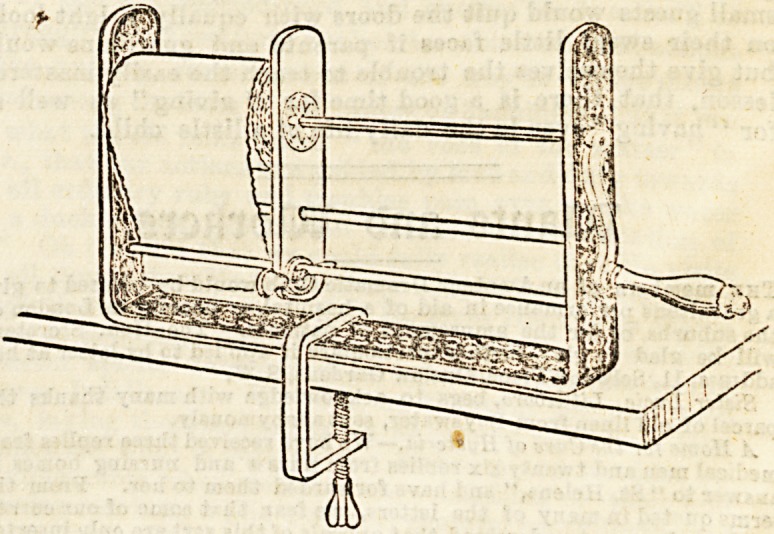


**Figure f4:**